# Supplementary material for: Chromothripsis during telomere crisis is independent of NHEJ, and consistent with a replicative origin
Source: Genome Res. 2019 May;29(5):737–49. doi: 10.1101/gr.240705.118 (PMC6499312; doi:10.1101/gr.240705.118)
Supplement: Supplemental Material [file supp_gr.240705.118_Supplemental_file_1.zip › contigs/annotated_contigs/DB71/contig.2.DB71_length_476_mean_cov_6.30672268908.docx]

**DB71_length_476_mean_cov_6.30672268908**

```
ATGTAACCCAGCATATTTACACATTTTGGGGATTAGGACATGGGTATCCTTGGGGGAATCAGCATTCAGCCTAACACAGTATATACTCT
 >chr5:21180643-21180776 + E=2e-68
ACATTTGGGGAAGTGGGTTTTCTATAAATGCTTTCTTGTTAAAG|CATA|TATATATATATATATATATATATATATATATATATATAT
                                                 >chr5:21183423-21183762 + E=1e-192
GCTCTTTGATGTTGATTTTGCTGTTTAAAATGGTCTAGTTGTCCTGCAGTAATAGCATTATGAATGGCTTAGAGTTTAGTAACATGAAC

AGTACCCTCAATTTTCTAAAACAAACAAAAAATATAGCTAATTAGTAAGCATAAGTAATGATAAAAGGCCAGAAATTAAGGATGATATT

AGCTAGGCTCTGTAAAAACTTTACTTCACTTTTTGCATCATTCAGAAAGAGGTTTGTGGGTTGGGCGCAGTGGCTCAAGCCTGTAATCC

CAGCACTTTGGGAGGCTGAGGTGGGGAGATCAC
```
